# Towards a new method for cardiac tissue velocity measurements using MRI, comparison with echocardiography

**DOI:** 10.1186/1532-429X-16-S1-P44

**Published:** 2014-01-16

**Authors:** Christophe Meyer, Pierre-André Vuissoz, Jacques Felblinger, Laurent Bonnemains

**Affiliations:** 1 IADI INSERM U947, Nancy, France; 2 IADI, Université de Lorraine, Nancy, France; 3 CHU Nancy, Nancy, France; 4 CIC-IT 801, INSERM, Nancy, France

## Background

Echocardiography is currently the gold standard for cardiac tissue velocity measurement [1]. Phase contrast MRI with multiple excitations averaging has been proposed as an alternative solution [2]. In this study, we used a central k-space line phase contrast sequence [3] to acquire real-time velocity curves and compared them to echocardiography.

## Methods

**Figure 1:**
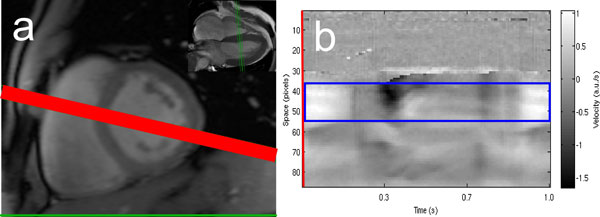
**(a) Placement of small axis plane (green) and frequency direction (red)**. (b) Time-space display of velocity data with automatically selected ROI (blue) corresponding to maximum spectral power in cardiac frequency band.

Eighteen healthy volunteers underwent cardiac MRI examination on a Signa HDxt 3T scanner (General Electric, Waukesha). A phase contrast sequence modified to acquire only the central k-space line was used in a mid-ventricular small axis scan plane with through-plane velocity encoding during 128 heartbeats. The frequency encoding direction was angulated to encompass the projection of the left ventricular outflow tract plane (Figure 1). Relevant parameters were: 256-point frequency k-space size, 35 cm FOV, 1 view per segment, 150 cm/s velocity encoding and 7 ms TR. Data were Fourier transformed into 1D+t image space, subjected to SVD decomposition for automatic spatial segmentation and finally automatic temporal domain peak detection. This process lead to the measurement of four durations for each heartbeat (RR): IsoVolumic Contraction (IVC), Systolic Ejection (SE), IsoVolumic Relaxation (IVR), and Diastolic Diastasis (DD). For every cardiac cycle, the results of the automatic detection were quality controlled by a physician who could either discard the data, correct or accept it. Myocardiac Performance Index (MPI) [4] was computed as (IVC+IVR)/SE as well as normalized systole duration (IVC+SE)/RR and normalized diastasis duration DD/RR. These values were checked against similar indices assessed with tissue Doppler echocardiography performed a few hours before the MRI.

## Results

**Figure 2:**
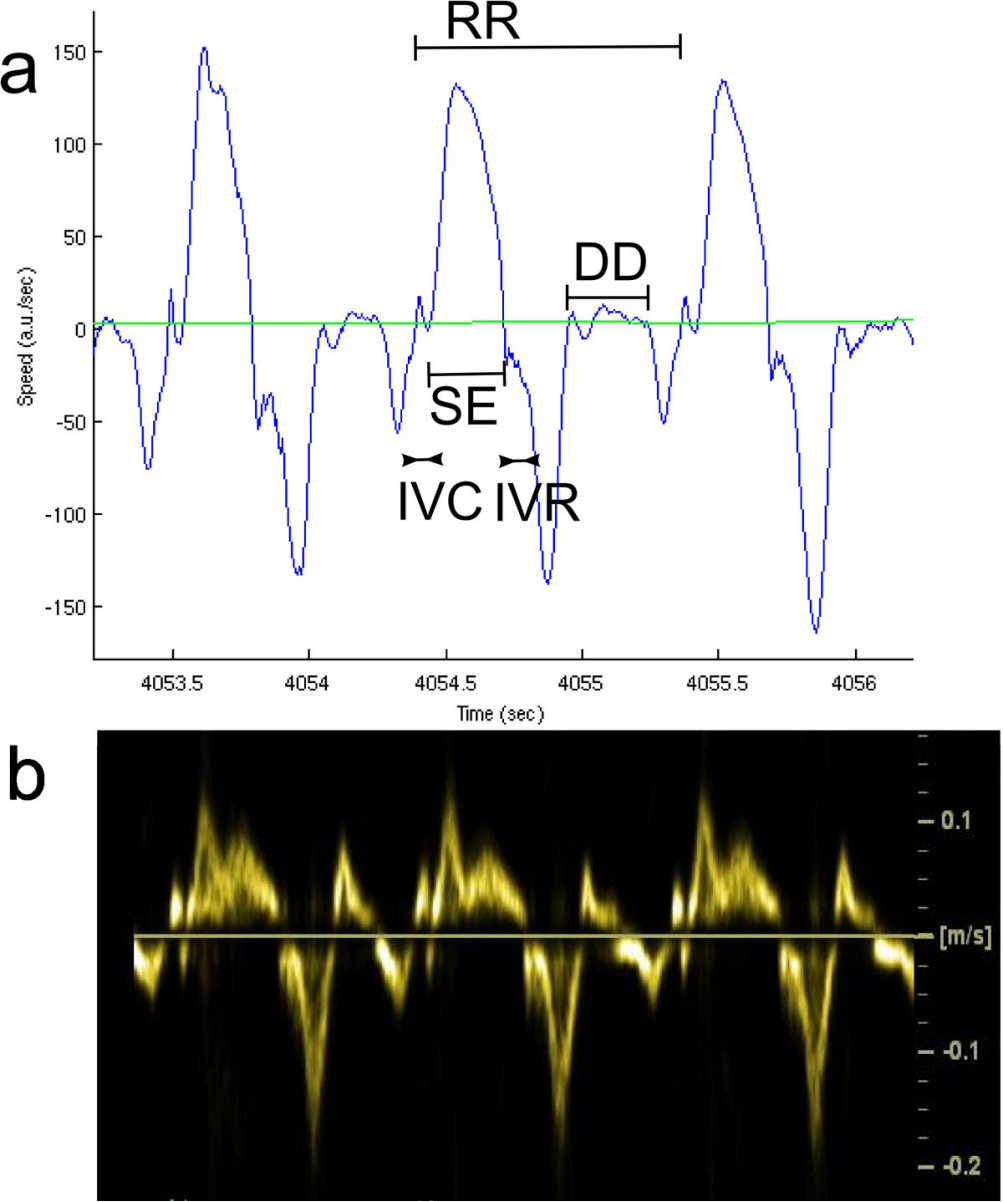
**(a) Example of velocity curve obtained from MRI computed as mean velocity in ROI of Figure 1 (blue)**. Cardiac cycle (RR), IsoVolumic Contraction (IVC), Systolic Ejection (SE), IsoVolumic Relaxation (IVR) and Diastolic Diastasis (DD) are depicted on the curve. (b) Similar velocity curve obtained from tissue Doppler echocardiography.

Seven subjects were discarded due to low echocardiographic or MRI data quality (impossibility to measure all the durations). One subject was manually post processed due to failure of automatic detection and a total of 360 heart beats were also discarded. For eleven subjects, velocity curves similar to echocardiographic ones were obtained (see Figure 2). Within the set of 1048 remaining heart beats, fair correlation was observed between echocardiographic values and the median of accepted MRI cardiac cycles: r-squared = 0.52 for MPI, 0.61 for systole and 0.74 for diastasis.

## Conclusions

This new method provides velocity curves very similar to echocardiographic tissue Doppler ones and the measurements of duration of the main cardiac cycle components (systole, diastasis) as well as MPI were well correlated. This paves the way for the observation of beat to beat intra-subject variability of cardiac indices.

## Funding

None.
